# 87. Refractory, Resistant and Recurrent Cytomegalovirus Infections in Solid Organ Transplant Recipients: Risk Factors and Clinical Outcomes

**DOI:** 10.1093/ofid/ofaf695.033

**Published:** 2026-01-11

**Authors:** Bismarck Bisono Garcia, Benjo P Ato, Christopher A Dinh, Vaisak O Nair, Zachary Yetmar, Raymund R Razonable

**Affiliations:** Mayo Clinic, Rochester, MN; Philippine General Hospital, Manila City, National Capital Region, Philippines; Mayo Clinic, Rochester, MN; Mayo Clinic, Rochester, MN; Cleveland Clinic, Cleveland, OH; Mayo Clinic, Rochester, MN

## Abstract

**Background:**

Cytomegalovirus (CMV) infection remains one of the most prevalent and important infections following solid organ transplant (SOT). Treatment is often challenging, especially when dealing with refractory and resistant CMV infections.

**Table 1:**
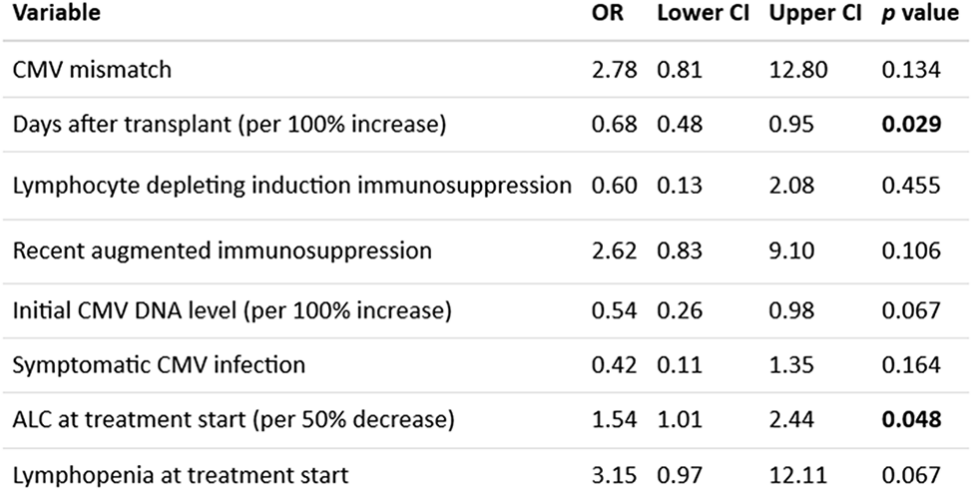
Univariable logistic regression analyses of associations with early refractory CMV infection

**Table 2:**
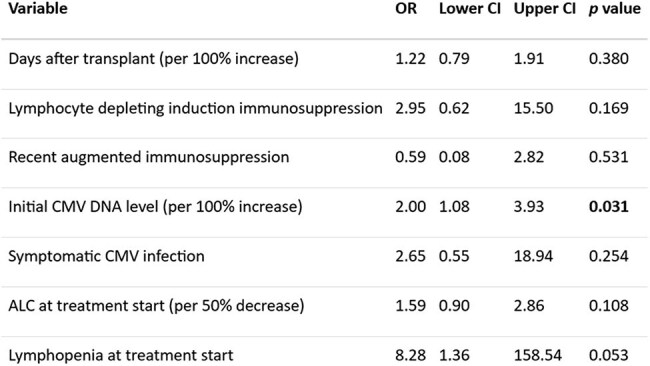
Univariable logistic regression analyses of associations with resistant CMV infection

**Methods:**

We performed a retrospective cohort study of SOT recipients with clinically significant CMV infection (csCMVi) at a tertiary transplant center in the US during 2010-2016. The primary outcome was early refractory CMV infection and secondary outcomes were drug resistance, CMV recurrence, and mortality. Kaplan-Meier estimation, univariable logistic regression analysis, and multivariable Cox regression were used for outcome analysis.

**Table 3:**
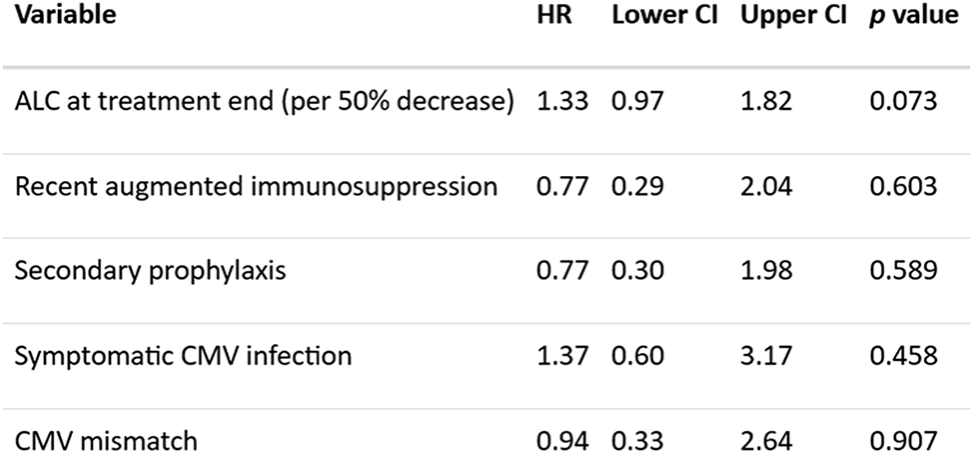
Multivariable Cox regression analysis of associations with recurrent CMV infection after primary treatment ended Note: Refractory CMV was not included in the model for recurrent CMV as no patients with refractory infection had recurrence.

**Results:**

A total of 145 SOT recipients with csCMVi were included. Most patients were recipients of liver (49%), followed by kidney (22.1%), heart (6.9%), lung (5.5%), and pancreas (4.1%). Multiple organ transplants accounted for 12.4% of the cohort. Most common induction was an anti-IL-2 antibody (43.9%) followed by ATG (29.5%). 82 (56.5%) had CMV D+/R- mismatch. In total, 34 (23.4%) had CMV syndrome, 38 (26.2%) had tissue-invasive CMV disease, while the rest (50.3%) were asymptomatic. After the initial 3 weeks of antiviral therapy, 13 (8.9%) patients had probable refractory csCMVi; most of them (10 [76.9%]) were CMV D+/R- mismatch. Longer time after transplant had lower risk (OR 0.68; CI 0.48-0.95, *p*=0.029) while lower absolute lymphocyte count (ALC) was higher risk of early refractory csCMVi (OR 1.54; CI 1.01-2.44, *p*=0.048) (Table 1). Drug-resistant CMV occurred in 7 patients (4.8%); a high initial CMV DNA level was associated with resistant csCMVi (OR 2; CI 1.08-3.93, *p*=0.031) (Table 2). Of 144 patients with post-treatment follow-up, 25 (17.2%) experienced recurrent csCMVi within 6 months. Table 3 shows the associations with recurrence of csCMVi after initial treatment. 30 patients (20.7%) died during a median follow-up of 4 years, but refractory, resistant or recurrent CMV were not associated with mortality.

**Conclusion:**

Refractory csCMVi is associated with low ALC and an earlier onset post-transplant. Resistant csCMVi is associated with high levels of initial CMV DNA. Recurrence of csCMVi is common and possibly associated with low ALC at the end of antiviral treatment.

**Disclosures:**

All Authors: No reported disclosures

